# Observation of nonlinear edge states in an interacting atomic trimer array

**DOI:** 10.1038/s41377-025-01997-6

**Published:** 2025-08-28

**Authors:** Huiying Du, Hongxing Zhao, Yuqing Li, Yunfei Wang, Rujiang Li, Jizhou Wu, Wenliang Liu, Yiqi Zhang, Liantuan Xiao, Suotang Jia, Jie Ma

**Affiliations:** 1 State Key Laboratory of Quantum Optics Technologies and Devices, Institute of Laser Spectroscopy, Shanxi University, Taiyuan, 030006 China; 2 Collaborative Innovation Center of Extreme Optics, Shanxi University, Taiyuan, 030006 China; 3 Hefei National Laboratory, Hefei, 230088 China; 4 Key Laboratory of Antennas and Microwave Technology, School of Electronic Engineering, Xidian University, Xi’an, 710071 China; 5 Key Laboratory for Physical Electronics and Devices, Ministry of Education, School of Electronic Science and Engineering, Xi’an Jiaotong University, Xi’an, 710049 China

**Keywords:** Atom optics, Solitons

## Abstract

Exploring the interplay between topology and nonlinearity leads to an emerging field of nonlinear topological physics, which extends the study of fascinating properties of topological states to a regime where interactions between the particles cannot be neglected. For ultracold atomic systems, although many exotic topological states have been recently observed, the nonlinear effect remains elusive. Here, based on the laser-driven couplings of discrete atomic momentum states, we synthesize a topological trimer array, where the atomic interactions give rise to tunable nonlinearities. We observe the formation of nonlinear edge states in the density population evolution and participation ratio with increasing interaction, in contrast to the diffusive transport in a broad interaction range in nontopological arrays. Furthermore, we show the impact of interactions on the population distribution evolved from the initialized single-site population. Our work opens the avenue for exploring emergent nonlinear topological behaviors in ultracold atomic gases.

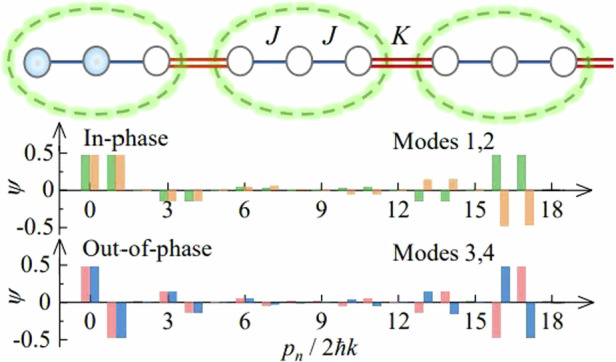

## Abstract (web summary)

We study the impact of interactions on the population distribution in a topological trimer array of ultracold atoms, and observe the formation of nonlinear edge states.

## Introduction

Topologically protected edge states emerging due to the formation of single-particle gap in nontrivial bulk bands are immune to local deformations and disorders, and offer promising opportunities for the design of desired optoelectronic devices^1–4^. Exploring the interplay between topological band structures and tunable nonlinearities in photonic lattices has led to many novel phenomena, such as lasing in topological edge states^5–7^, harmonic generation^8,9^, edge solitons^10–14^ and bulk solitons in topological bandgaps^15^. The quantized transport and fractionally quantized motion of solitons in nonlinear Thouless pumps are reported recently^16–20^, raising the hope to engineer the topologically quantized and fractional transports in synthetic nonlinear systems. Progress in this emerging field of nonlinear topological physics opens intriguing prospects for studying the novel strongly correlated states of light and controlling its behavior^21–23^.

On the other hand, the high level of control over ultracold atomic systems has enabled the observation of several topological features, including topological Chern invariants^24,25^, Hall drift^26^, Berry curvature^27,28^, edge states^29,30^ and quantized pumping^31,32^. In particular, the recently developed momentum-lattice technique further facilitates the realization of paradigmatic one-dimensional (1D) Su-Schrieffer-Heeger (SSH) model with dimerized lattice hoppings^33,34^, and the topological edge state, Anderson insulator and quantum walk were observed in a site-resolved manner^35–39^. Moreover, momentum lattices also provide an efficient avenue for exploring the nonlinear effect on the quantum transport^40–45^ and finding the beyond-mean-field behavior^46^, where the atomic interactions give rise to the nonlinearity. However, the interplay between topology and tunable nonlinearity is still elusive.

In this Letter, we experimentally synthesized a topological trimer array by adopting the momentum-lattice technique in a ^133^Cs Bose-Einstein condensate (BEC), where the atomic interactions controlled via a broad Feshbach resonance lead to tunable nonlinearities. Combined with the precise control of the lattice parameters, we study the interplay between topology and nonlinearity by measuring the effect of varying interactions on the density population and participation ratio in the quench dynamics of topological atomic trimer arrays. When our system is initialized at two types of edge states emerging in two topological band gaps, we observe the formation of nonlinear edge states characterized by the localization of all atoms on two boundary sites with increasing interaction, whereas they cannot be formed in a broad interaction range in nontopological arrays. For the single-site injection, we observe that the population distribution obtained after an evolution time is consistent with that determined by the topological edge states in noninteracting or weakly interacting regimes, while all atoms become localized at the initial site for large interactions.

## Results

### Implementation of topological trimer array

**Fig. 1:**
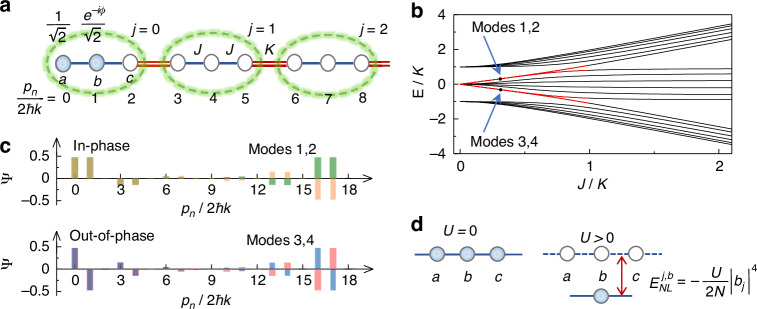
Synthesizing topological atomic trimer array with tunable interaction. **a** A topological trimer array is implemented in a momentum lattice, based on the laser-driven Bragg transitions between the discrete atomic momentum states *p*_*n*_ with *n* ∈ {0,17}. Each unit cell in the trimer array includes three sites (*a*, *b*, *c*), and the unit cell index *j* ranges from 0 to 5. The intra- and intercell hopping rates are characterized by *J* and *K*, respectively, and can be individually controlled by addressing each Bragg transition. Atoms are prepared at two leftmost sites with equal weight and a relative phase *ϕ*. **b** Energy spectra as a function of *J/K* in the trimer array with open boundary condition in the noninteracting limit. The red lines represent topological edge states for *J/K* < 1. **c** Eigenmodes of topological trimer array correspond to the black dots in (**b**). Two in-phase (out-of-phase) peaks characterize the modes 1 and 2 (modes 3 and 4). **d** Illustration of the density-dependent, nonlinear energy induced by the atomic interactions. The site energy is shifted by ${E}_{{NL}}^{j,b}=-{{U|}{b}_{j}|}^{4}/2N$ with the mean-field interaction energy *U* and atomic population |*b*_*j*_|^2^ at site |*j*,*b*〉

For ultracold atoms in a trimer array with three sites (*a*, *b*, *c*) per unit cell as depicted in Fig. 1a, the system can be described by an extended 1D SSH model^47^, where *j* is the index of unit cell, and the intra- and intercell hopping rates are characterized by *J* and *K*, respectively. We experimentally implement the trimer array in a momentum lattice of ^133^Cs BEC with *N* = 4 × 10^4^ atoms^48^. A pair of counter-propagating laser beams with wavelength *λ* = 1064 nm are used to illuminate the BEC for driving a series of two-photon Bragg transitions between discrete atomic momentum states *p*_*n*_ = 2*nħk* with the reduced Planck’s constant *ħ* and wave vector *k* = 2π/λ^49–51^. The 1D momentum lattice is synthesized by denoting momentum states as lattice sites (see Fig. 1a), and the hopping rates are precisely controlled through the distinct Bragg transitions [see “Material and methods”]. A topological trimer array consisting of 6 unit cells is formed by controlling the hopping rates in a 18-site momentum lattice in a trimer structure.

In the absence of interactions, the dynamics of atoms in a trimer array is governed by the Hamiltonian

1 $$H=\sum _{j}(J{b}_{j}^{\dagger }{a}_{j}+J{c}_{j}^{\dagger }{b}_{j}+K{a}_{j+1}^{\dagger }{c}_{j}+H.c.)$$

where ${a}_{j}^{\dagger }$(${a}_{j}$), ${b}_{j}^{\dagger }$(${b}_{j}$) and ${c}_{j}^{\dagger }$(${c}_{j}$) are, respectively, the creation (annihilation) operators for sites *a*, *b* and *c* of the *j*th unit cell, and we denote the corresponding states as |*j*, *a*〉, |*j*, *b*〉 and |*j*, *c*〉. When the ratio of the intra- and intercell hopping rates is tuned to *J*/*K* < 1, and the system is driven into topological phase^14,47^. For a standard SSH model in the dimer array^35–39^, there is a single topological band gap in the energy spectrum, allows to form only one pair of edge states. However, the energy spectrum of the trimer array exhibits two topological band gaps, where two pairs of edge states appear with different internal structure as shown in Fig. 1b. Two edge states in each gap become nearly degenerate with decreasing *J*/*K*. In Fig. 1c, we show the wavefunctions of topological edge states for *J*/*K* = 0.31, corresponds to the eigenmodes 1–4 indicated in Fig. 1b. Two in-phase peaks in two leftmost sites characterize the symmetric modes 1 and 2 in the top gap, while two out-of-phase peaks characterize the antisymmetric modes 3 and 4 in the bottom gap^14^. The bulk topological invariants corresponding to the topological edge states are characterized by the quantized Zak phase with periodic boundary condition. The Zak phases of the top and bottom bands are *π* if *J*/*K* < 1 and 0 if *J*/*K* > 1. For the middle band, the Zak phase is 2*π* if *J*/*K* < 1 and 0 if *J*/*K* > 1 (see Supplementary Information).

To provide an insight into the tunable nonlinearity induced by the atomic interactions, we follow the widely adopted mean-field approximation in momentum lattices^40–45^, and write down the nonlinear Hamiltonian

2 $${H}_{NL}=U\left(N-\frac{1}{2}\right)-\frac{U}{2N}\sum _{j}\left({|{a}_{j}|}^{4}+{|{b}_{j}|}^{4}+{|{c}_{j}|}^{4}\right)$$

where *N* is the total atom number and the mean-field interaction energy *U* is proportional to the atomic s-wave scattering length *a*_s_^52,53^. By neglecting the irrelevant energy shift *U*(*N* − 1/2), the interactions with *a*_s_ > 0 lead to a density-dependent nonlinear energy in Fig. 1d. Different from the Kerr nonlinearity in optical systems^14,54–57^, the nonlinearity induced by the interactions can be directly tuned through a broad Feshbach resonance of ^133^Cs BEC^53^, which provides an effective avenue to explore nonlinear topological behavior in ultracold atoms. By combing the single-particle Hamiltonian (1) and the nonlinear Hamiltonian (2), we can perform the numerical simulation for the dynamics of atoms in the trimer array under different interactions.

In the presence of interactions, the theoretical calculations show that there is a variety of nonlinear edge states for a fixed *U*/*K*, and the density of the state changes successively with the energy *E* (see Supplementary Information). However, we consider only two kinds of nonlinear edge states that bifurcate from the in-phase and out-of-phase linear edge states, because these states are not only stable and robust (see Supplementary Information) but also experimentally excited by engineering the initial population^14^. Also, as shown in the Supplementary Information, the nonlinear edge states reduce into their linear counterparts if their density decreases successively with the energy *E*, which means that they bifurcate from and therefore inherit the topological properties from their linear counterparts. As a result, these nonlinear edge states are still topologically protected as long as the corresponding propagation constant (i.e., energy *E*) is still in the band gap^58,59^. Thus, the energy spectra and the corresponding topological invariant obtained in noninteracting limit are crucial for analyzing the interaction-induced nonlinear edge states.

### Observation of nonlinear edge states

The nonlinear edge states bifurcating from the in- and out-of-phase linear edge states determine that the atoms are more localized at two leftmost sites in the topological trimer array for strong *U*/*K*. Taking advantages of the site-resolved control over the hopping rates and tunneling phases in momentum lattices, we engineer the initial in- and out-of-phase population at two leftmost sites, and then study the impact of atomic interactions on the population distribution after the quench to the topological trimer array. We begin with all hoppings turned off, and all atoms are prepared at a single site |0, *a*〉. Due to the decoherence of the BEC from the physical separation of wave packets with different momenta, it is difficult to prepare the initial population at two boundary sides with controlled proportion following the distribution determined by the nonlinear edge states. We use a π/2 Bragg pulse to create a superposition state with the equal occupation on two sites |0, *a*〉 and |0, *b*〉 (see Fig. 1a). By imposing the phase *ϕ* in the corresponding Bragg transition, we initialize the atoms at two leftmost sites with a relative phase of *ϕ*, and hope such an initialization can be approximate to the distribution demanded by the nonlinear edge states.

The initialized population is then quenched to a topological trimer array for *J*/*K* = 0.31 under different interactions *U*/*K*, and the population distribution is measured after an evolution time *t* = 3.2*ħ*/*K* by using the absorption image following 22 ms time-of-flight (TOF). We use the atomic population at two leftmost sites *P*_s_ = |*a*_0_|^2^ + |*b*_0_|^2^ to characterize the impact from interactions. We further define the participation ratio to characterize the localization degree of atoms in the array

3 $$r=\frac{1}{L}\frac{1}{{\sum }_{j}\left({|{a}_{j}|}^{4}+{|{b}_{j}|}^{4}+{|{c}_{j}|}^{4}\right)}$$

where *L* is the lattice size. For a diffusive distribution, the maximum possible *r* is 1, and it decreases with enhanced localization. For *L* = 18, the localization of all atoms at two sites with the equal weight is characterized with *r* = 0.11, and the single-site localization has *r* = 0.055.

**Fig. 2:**
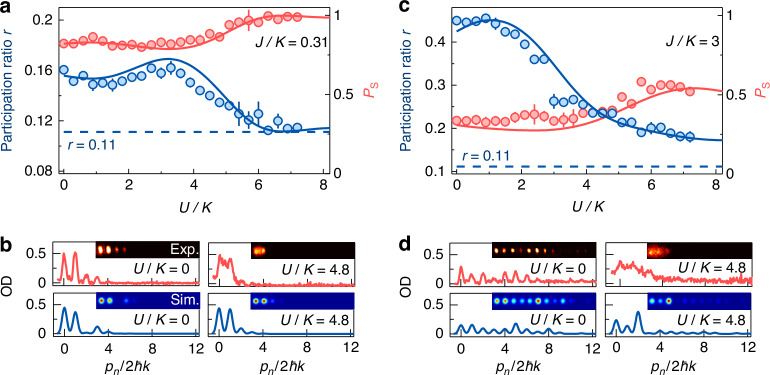
Interaction-induced nonlinear edge state for the initialized in-phase population. **a** Atomic population in two leftmost sites *P*_s_ (red circles) and participation ratio *r* (blue circles) in a topological trimer array for *J/K* = 0.31 as a function of interaction *U/K*. **b** Population distributions in the topological trimer array with *J/K* = 0.31 for *U/K* = 0 and 4.8. **c** Variations of *P*_s_ (red circles) and *r* (blue circles) in a nontopological array for *J/K* = 3 with *U/K*. **d** Population distributions in the nontopological array with *J/K* = 3 for *U/K* = 0 and 4.8. The data in (**a**, **c**) are obtained after the time *t* = 3.2*ħ/K*, and the solid lines are the numerical simulations. The images in (**b**, **d**) are taken after *t* = 3.2*ħ/K* evolution and 22 ms TOF. All error bars denote standard errors. In all panels, the atoms are equally initialized at two leftmost sites with phase *ϕ* = 0, and the intercell hopping rate is *K/h* = 0.4 kHz

In Fig. 2a, the population at two leftmost sites *P*_s_ and participation ratio *r* are plotted as a function of *U*/*K* for *J*/*K* = 0.31 and *ϕ* = 0. In the noninteracting and weakly interacting regimes, the initial population is coupled to the in-phase topological modes, and the atoms are mainly populated at two leftmost sites. However, a small number of atoms are diffused to other sites with *P*_s_ < 1 and *r* > 0.11. *P*_s_ and *r* increases and decreases, respectively, with increasing *U*/*K*, and the atoms are largely localized at two leftmost sites. The typical atomic distributions for *U*/*K* = 0 and 4.8 are shown in Fig. 2b. When *U*/*K* is increased to strong enough, all atoms are localized at two leftmost sites with *P*_s_ = 1 and *r* = 0.11, in consistent with the self-trapping^60–62^. This qualitatively agrees with the localization of more atoms at two leftmost sites, which is determined by the nonlinear edge states under the strong interactions [14, see also Supplementary Information].

For a nontopological array with *J*/*K* = 3, the variations of *P*_s_ and *r* with *U*/*K* are shown in Fig. 2c. A large number of atoms initialized at two leftmost sites with *ϕ* = 0 are diffused to other sites in a broad range of *U*/*K*, and even we have *P*_s_ ≤ 0.5 and *r* > 0.11 for strong *U*/*K*. In Fig. 2d, we show the atomic distributions in the nontopological array for *U*/*K* = 0 and 4.8. The data in Fig. 2 are in good agreement with the numerical simulations. The blurred profiles in Fig. 2b, d are likely caused by the thermalization of the BEC under the strong interactions.

**Fig. 3:**
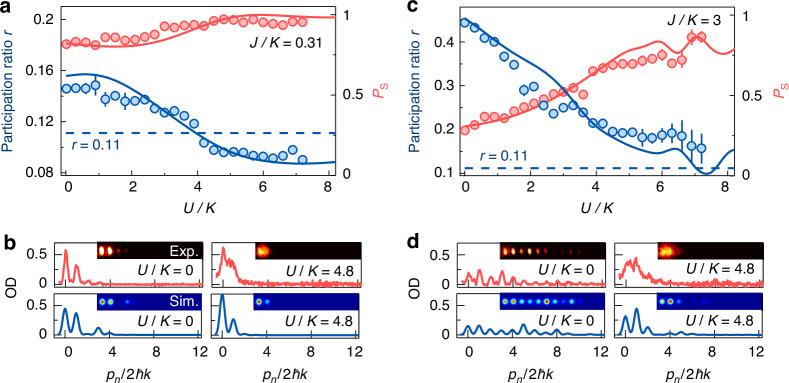
Interaction-induced nonlinear edge state for the initialized out-of-phase population. **a** Atomic population in two leftmost sites *P*_s_ (red circles) and participation ratio *r* (blue circles) in a topological trimer array for *J/K* = 0.31 as a function of interaction *U/K*. **b** Population distributions in the topological trimer array with *J/K* = 0.31 for *U/K* = 0 and 4.8. **c** Variations of *P*_s_ (red circles) and *r* (blue circles) in a nontopological array for *J/K* = 3 with *U/K*. **d** Population distributions in the nontopological array with *J/K* = 3 for *U/K* = 0 and 4.8. The data in (**a**, **c)** are obtained after the time *t* = 3.2*ħ/K*, and the solid lines are the numerical simulations. The images in (**b**, **d**) are taken after *t* = 3.2*ħ/K* evolution and 22 ms TOF. All error bars denote standard errors. In all panels, the atoms are equally initialized at two leftmost sites with phase *ϕ* = π, and the intercell hopping rate is *K/h* = 0.4 kHz

In Fig. 3a, we show the variations of *P*_s_ and *r* with *U*/*K* for *J*/*K* = 0.31 and *ϕ* = π. We observe the localization of the atoms at two leftmost sites under the strong interactions, where the self-trapping occurs^60–62^. Different from the equal occupation for *ϕ* = 0, the atomic population at two leftmost sites becomes unequal with *r* < 0.11, for example, the atomic distribution for *U*/*K* = 4.8 in Fig. 3b. This unequal population results from the oscillation in the measured population dynamics (see Supplementary Information). The numerical calculation of population dynamics at the long time shows the unstable oscillation and population imbalance at two leftmost sites (see Supplementary Information), which illustrates that the initial out-of-phase population cannot be approximate to the distribution determined by the nonlinear edge state, in contrast to the initial in-phase population. Although the stationary states are not reached for *ϕ* = π, the enhanced localization at two leftmost sites observed in the population dynamics illustrates that the initial population is approaching to the distribution determined by the nonlinear state under the strong interactions rather than the cases for noninteraction or weak interactions. For a nontopological array with *J/K* = 3, the diffusive distribution is observed in a large range of *U/K* in Fig. 3c. The population distributions of atoms in the nontopological array for *U/K* = 0 and 4.8 are shown in Fig. 3d. The data in Fig. 3 are in good agreement with the numerical simulations.

### Interaction effect on the atomic population distribution evolved from the initialized single-site population

**Fig. 4:**
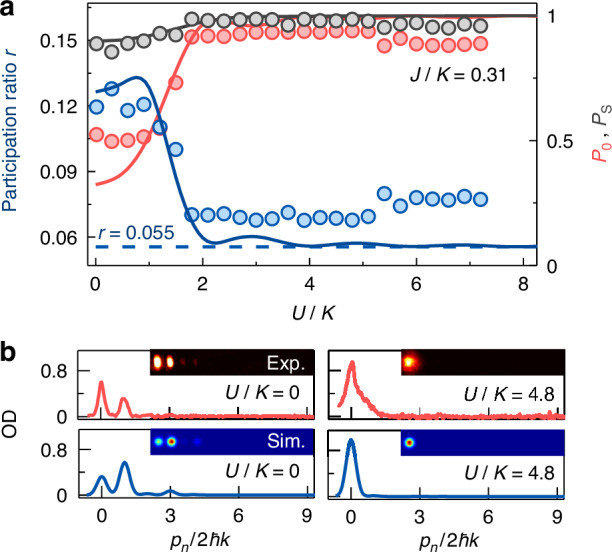
Interaction effect on the population distribution evolved from the initialized single-site population. **a** Atomic populations in one and two leftmost sites *P*_0_ (red circles) and *P*_s_ (gray circles) as well as the participation ratio *r* (blue circles) in a topological trimer array for *J/K* = 0.31 obtained after an evolution time *t* = 3.2*ħ/K* as a function of interaction *U/K*. The solid lines are the numerical simulations, and all error bars denote standard errors. **b** Population distributions in the topological trimer array for *U/K* = 0 and 4.8. In all panels, the atoms are initialized at the leftmost site, and the intercell hopping rate is *K/h* = 0.4 kHz

Different from the preparation of initial population at two leftmost sites, we directly quench all atoms initialized at the leftmost site |0, *a*〉 to a topological trimer array with *J/K* = 0.31 for different *U/K*. The population distribution is measured after an evolution time *t* = 3.2*ħ/K*, and the variations of *P*_0_, *P*_s_ and *r* with *U/K* are shown in Fig. 4a, where *P*_0_ is the population of atoms in the leftmost site. In the noninteracting and weakly interacting regimes, we have *P*_s_ ∼ 0.9 and *r* ∼ 0.11, indicating that the initialized single-site population is evolved to a state, whose population distribution is consistent with that determined by the in- or out-of-phase topological edge states. In Fig. 4b, all atoms are almost populated at two leftmost sites for *U/K* = 0. When *U/K* is largely increased, we observe that all atoms become localized at the leftmost site with *P*_0_ = 1 and *r* = 0.055 in Fig. 4a, and the self-trapping occurs under the strong interaction^60–62^. Our observation indicates that the interactions affect the population distribution evolved from the initialized single-site population. The localization of atoms at the leftmost site for *U/K* = 4.8 is shown in Fig. 4b. The experiment agrees qualitatively with the numerical simulation.

## Discussion

In conclusion, we report the first experimental observation of nonlinear edge states in a topological trimer array of interacting ultracold atoms. Our work provides a starting point for the study of nonlinear topological physics in ultracold atomic systems, which extends the domain of nonlinear topological photonics. Because the nonlinearity is stemmed from the atomic interactions, our results offer intriguing insights for understanding the interplay between topology and interaction. In future, the ability of local and time-dependent control over hopping rates and tunneling phases in momentum lattices allow to probe the phase involved in two types of nonlinear edge states.

## Material and methods

### Synthesizing a trimer array

Our experiment starts with a ^133^Cs BEC in the hyperfine state $|F=3,{m}_{F}=3\rangle$ in a cigar-shaped optical trap. The laser beam, which provides the strong radial confinement, is retroreflected to form a pair of counter-propagating laser beams for illuminating the optically trapped BEC. We imprint the multiple-frequency components on the reflected laser beam to drive 17 different two-photon Bragg transitions between the discrete momentum states with an increment of 2*ħk*, and these laser-driven couplings synthesize a 1D momentum lattice. According to the quadratic energy-momentum dispersion relation, every Bragg transition has a unique frequency difference. Thus, we can address the strength of each Bragg transition by controlling the power of the corresponding frequency component, and all the hopping rates can be individually controlled. When the hopping rates are modulated in a trimer structure, we synthesize a trimer array in momentum space. The two-site Rabi oscillations are frequently implemented for calibrating the intra- and intercell hopping rates in the trimer array.

### Interaction-induced nonlinearity in momentum lattices

Different from the two-body contact interactions in real space, the atomic interactions become long-ranged in momentum space. Under the mean-field approximation, the effect of interactions on the transport dynamics of atoms in momentum lattices can be captured by a nonlinear Hamiltonian (see Eq. (2) in the main text), as described in refs. ^40–45^. By neglecting the irrelevant energy shift, the density-dependent nonlinear energy is proportional to the mean-field interaction energy $U=(4\pi {\hslash }^{2}{a}_{s}/m)\rho$, where $\rho =4\times {10}^{13}{{\rm{cm}}}^{-3}$ is the averaged atomic density with the local density approximation, *a*_*s*_ is the *s*-wave scattering length of ^133^Cs atoms, and *m* is the atomic mass. In our experiment, the scattering length *a*_*s*_ can be widely tuned through a broad Feshbach resonance^54^, which enables the tunable nonlinearity in a wide range.

### Experimental measurement

To study the effect of interaction on the density population evolution and participation ratio in a topological trimer array in momentum space, we quench the initialized atom population to the topological trimer array under various interactions, and measure the effect of interactions on the population distribution after an evolution time. We switch off all laser fields and quickly tune the uniform magnetic field to 17 G with zero-crossing scattering length, and measure the populations of atoms at different momentum states by taking an absorption image after 22 ms TOF. Since the atoms in the distinct momentum states have different momenta, they separate during the TOF. This allows us to measure the population distribution of atoms in the trimer array in a site-resolved manner.

## Supplementary information

Supplementary Information for ''Observation of nonlinear edge states in an interacting atomic trimer array''

## Data Availability

All experimental data and any related experimental background information not mentioned in the text are available from the authors upon reasonable request.
