# Supplementary material for: Observation of nonlinear edge states in an interacting atomic trimer array
Source: Light Sci Appl. 2025 Aug 28;14:296. doi: 10.1038/s41377-025-01997-6 (PMC12394654; doi:10.1038/s41377-025-01997-6)
Supplement: Supplementary file 1 — Supplementary Information for ''Observation of nonlinear edge states in an interacting atomic trimer array'' [file 41377_2025_1997_MOESM1_ESM.pdf]

Supplementary Information for "Observation of nonlinear edge states in an interacting atomic trimer array"

Huiying Du[1], Hongxing Zhao[1], Yuqing Li[1,2,3], Yunfei Wang[1], Rujiang Li[4], Jizhou Wu[1,2], Wenliang Liu[1,2], Yiqi Zhang[5], Liantuan Xiao[1,2], Suotang Jia[1,2], and Jie Ma[1,2,3]

[1]State Key Laboratory of Quantum Optics Technologies and Devices, Institute of Laser Spectroscopy, Shanxi University, Taiyuan 030006, China
[2]Collaborative Innovation Center of Extreme Optics, Shanxi University, Taiyuan 030006, China
[3]Hefei National Laboratory, Hefei 230088, China
[4]Key Laboratory of Antennas and Microwave Technology, School of Electronic Engineering, Xidian University, Xi'an 710071, China
[5]Key Laboratory for Physical Electronics and Devices, Ministry of Education, School of Electronic Science and Engineering, Xi'an Jiaotong University, Xi'an 710049, China

In the Supplemental Material, we provide more details on the energy spectra and eigenmodes of a topological trimer array with large size, the topological analysis of trimer array, the measured population dynamics in topologically nontrivial and trivial arrays, the numerical calculation of population dynamics at the long time, the nonlinear edge state families that bifurcate from their linear counterparts, and the stability and robustness of the nonlinear edge states.

Energy spectra and eigenmodes of a topological trimer array with large size

In this section, we show the energy spectra and eigenmodes of a topological trimer array with a size larger than the experiment in the noninteracting regime. In Fig. S1a, we show the energy spectra of a trimer array including 30 sites, and two pairs of edge states appear in each of two topological band gaps. In Fig. S1b, we show the wavefunctions of topological edge states for $J/K = 0.31$, corresponds to eigenmodes 1-4 indicated in Fig. S1a. In compared to the results in Fig. 1b and c in the main text, we see that the size effect does not qualitatively change the topological edge states and the corresponding eigenmodes.

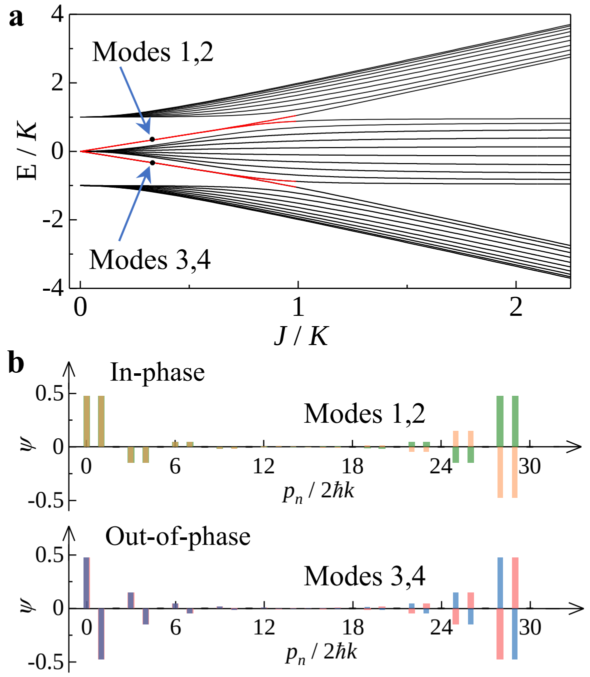

FIG. S1. **a**, Energy spectra as a function of the intra- to intercell hopping rate ratio J/K in the trimer array consisting of 30 sites with open boundary condition in the noninteracting limit. The red lines represent topological edge states for $J/K < 1$. **b**, Eigenmodes of topological trimer array for $J/K = 0.31$ correspond to the black dots in **a**.

Topological analysis of the trimer array

In the absence of interaction, the topological property of the trimer array is analyzed by checking the Zak phase γ of each band as a function of J/K. Since the Zak phase is an invariant of the bulk band, we consider the Hamiltonian of the trimer array, that can be written as

$$H_{\text{bulk}} = \begin{bmatrix} 0 & J & K\exp(-i\kappa) \\ J & 0 & J \\ K\exp(i\kappa) & J & 0 \end{bmatrix}. \tag{S1}$$

Here, we assume the separation between two nearest neighbor sites is 1, and the width of the first Brillouin zone (BZ) is $\kappa = 2\pi$. If the eigenstate of one band of the Hamiltonian H_{bulk} is labels as $|u(\kappa)\rangle$, the Zak phase is given by

$$\gamma = i\int_{\text{BZ}} \langle u(\kappa)|\partial_\kappa|u(\kappa)\rangle d\kappa. \tag{S2}$$

In Fig. S2a, we show the band structure of the trimer array in the first Brillouin zone with increasing J/K. We can see the band gaps close at $J/K = 1$, and open again if J/K increases further. To see the dependence of the band structure E/K on J/K, we project the band structure on the plane (J, E) in Fig. S2b, and the profile is the same as that of the energy spectra shown in Fig. 1b of the main text. In Fig. S2c, we show the variation of Zak phases of the bands with J/K, and the atomic trimer array indeed undergoes a topological phase transition. The Zak phases of the first

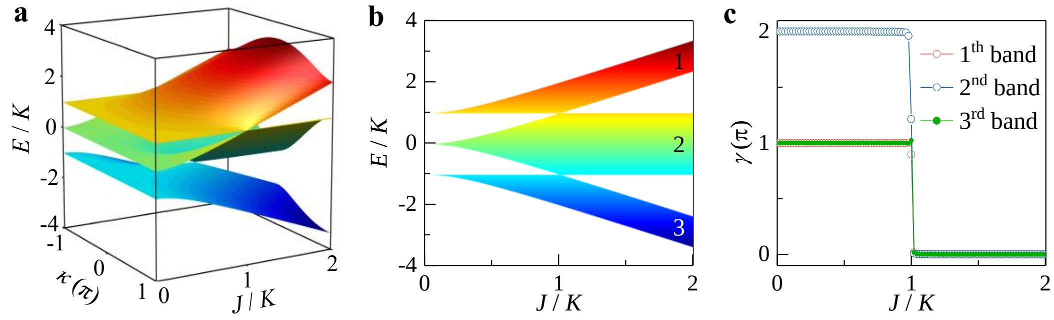

FIG. S2. **a**, Band structure of the trimer array in the first Brillouin zone $-\pi \leq \kappa \leq \pi$ as a function of the intra- to intercell hopping rate ratio J/K. **b**, Dependence of band structure on J/K by projecting the band structure in **a** on the (E, J) plane. **c**, The Zak phase of each band of the trimer array as a function of J/K.

(red circles) and third (green dots) bands are $\gamma = \pi$ in the region $J/K < 1$, and it becomes 0 if $J/K > 1$. Since the Zak phase of the second band is 2π in $J/K < 1$ and 0 in $J/K > 1$, the second band does not affect the topological property of the two band gaps. As a result, the topological edge states emerge in the both two band gaps, as shown in Fig. 1b in the main text or in Fig. S1a.

Measured population dynamics in topologically nontrivial and trivial arrays

In this section, we measure the dynamics of atomic populations in the topological and nontopological arrays for different interactions U/K, where the atoms are initialized at two leftmost sites with equal weight. In Fig. S3a-c, we show the dynamics of atomic populations at one and two leftmost sites P_0 and P_S after the quench to a topological trimer array with $J/K = 0.31$ under different interactions for the initial phase $\phi = 0$. We observe the almost equal occupation of atoms on two leftmost sites within the time $t = 6\hbar/K$ for $U/K = 6$ and 7.2, where the evolution time is limited by the decoherence of the BEC from the physical separation of wave packets with different momenta. In the nontopological array with $J/K = 3$, the atoms are mainly localized in the leftmost cell for $\phi = 0$ in Fig. S3d-f, but the strong intracell hopping rate induces the oscillation dynamics.

We show the dynamics of atomic populations P_0 and P_S in the topological trimer array with the initial phase $\phi = \pi$ for different U/K in Fig. S4a-c. The atoms populating at two leftmost sites have the unstable oscillation, which leads to the unequal population observed after the experimental duration $t = 3.2\hbar/K$ with $r < 0.11$ under the strong interactions in Fig. 3a of the main text. In the nontopological array with $\phi = \pi$, we observe the oscillation dynamics of atoms in the leftmost cell in Fig. S4d-f.

Numerical calculation of population dynamics at the long time

In principle, if the initialized population is closely approximate to the theoretical distribution from the nonlinear edge state under the strong interactions, the stable population dynamics should be observed to support the stationary state. However, the measured dynamics in Fig. S3a-c cannot verify whether the unstable oscillations occur at long times for $\phi = 0$, and the oscillation dynamics are observed for $\phi = \pi$ in Fig. S4a-c.

To confirm whether the stationary states occur at the long time, we perform the numerical calculation to show the population dynamics at the long time in Fig. S5, where the initial population is the same as the experimental initialization. For $\phi = 0$, the numerical population dynamics show that the atoms always populate at two leftmost sites with the equal weight for $U/K = 6$, i.e., the stationary state exists, but the unstable oscillations for $U/K = 4.8$ and 7.2 illustrate that the system is evolved to the non-stationary states. For $\phi = \pi$, the unstable dynamics with the small oscillation amplitudes for $U/K = 4.8$, 6 and 7.2 illustrate that there is not stationary state, and even the large population imbalance between the two leftmost sites emerges at the long time. Both the unstable oscillation and population imbalance illustrate that the initialized out-of-phase population cannot be approximate to the distribution determined by the corresponding nonlinear edge state.

We further define the averaged deviation from the mean population to quantitatively characterize the amplitude of oscillation

$$\Delta P_n = \frac{1}{t_2 - t_1} \int_{t_1}^{t_2} \left| P_n(t) - \overline{P}_n \right| dt, \qquad \text{(S3)}$$

where P_n is the atomic population at site n, and the corresponding mean population is given as $\overline{P}_n = \frac{1}{t_2 - t_1} \int_{t_1}^{t_2} P_n(t) dt$. We calculate the variations of ΔP_0 and ΔP_1 with U/K, and the average is performed over the time range from $t_1 = 20\hbar/K$ to $t_2 = 60\hbar/K$. The numerical results in Fig. S5d clearly demonstrate that the stationary states exist under most of the strong interactions for $\phi = 0$, and the non-stationary states emerging under the interactions ranging from $U/K = 6.6$ to 7.8 may be attributed to that the initial in-phase population cannot be approximate to the distributions determined by the eigenmodes of nonlinear edge states. For $\phi = \pi$, the oscillation dynamics at the long time lead to the non-zero ΔP_0 and ΔP_1 under the strong interactions

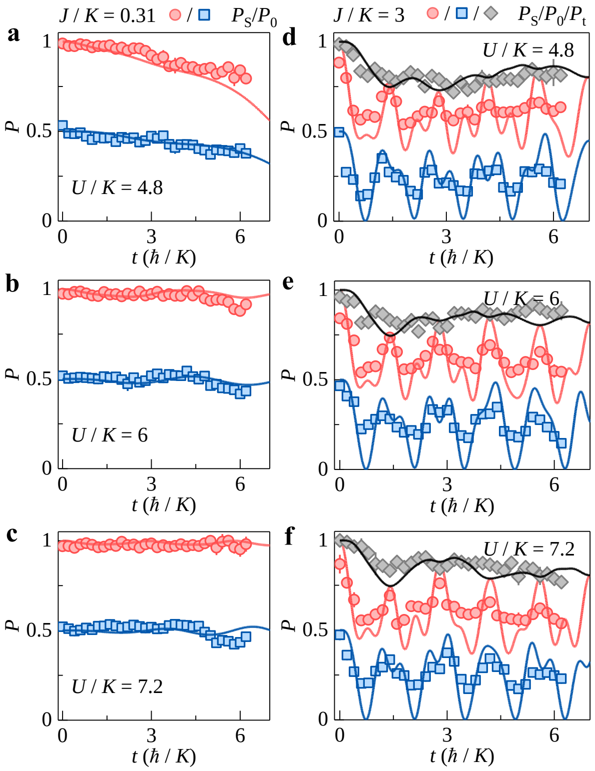

FIG. S3. Measured population dynamics of atoms in the topologically nontrivial and trivial arrays under different interactions for the initial in-phase population. **a**-**c**, Dynamics of atomic populations at one and two leftmost sites P_0 and P_S in a topological trimer array with $J/K = 0.31$ for different U/K. **d**-**f**, Dynamics of atomic populations at one, two and three leftmost sites P_0, P_S and P_t in a nontopological array with $J/K = 3$ for different U/K. The solid lines are the numerical simulations. All error bars denote standard errors. In all panels, the atoms are equally initialized at two leftmost sites with phase $\phi = 0$, and the intercell hopping rate is $K/h = 0.4\,\mathrm{kHz}$.

in Fig. S5h, and this illustrates that there is not stationary state.

In theory, the nonlinear edge states that bifurcate from both the in-phase and out-of-phase linear edgestates are stable, as demonstrated by the following linear stability analysis. When the initialized population is completely consistent with the distribution determined by the eigenmodes of the nonlinear edge state for a fixed interaction, the populatlon distribution remains unchanged in the dynamic evolution process, i.e., the stationary state is reached. In the experiment, limited by the decoherence from the spatial separation of atoms populating at different momentum states, it is very difficult to initialize the atomic populations at several distinct momentum states precisely in accordance with the eigenmodes of the nonlinear edge states. However, theoretically, the nonlinear edge states determine the localization of most atoms at two leftmost sites under the strong interactions. Thus, the atoms are initialized at two leftmost sites with the

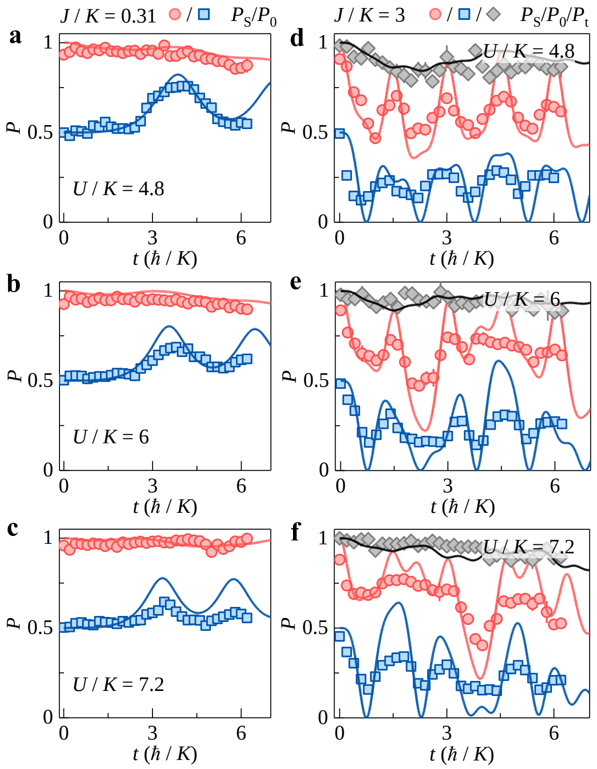

FIG. S4. Measured population dynamics of atoms in the topologically nontrivial and trivial arrays under different interactions for the initial out-of-phase population. **a**-**c**, Dynamics of atomic populations at one and two leftmost sites P_0 and P_S in a topological trimer array with $J/K = 0.31$ for different U/K. **d**-**f**, Dynamics of atomic populations at one, two and three leftmost sites P_0, P_S and P_t in a nontopological array with $J/K = 3$ for different U/K. The solid lines are the numerical simulations. All error bars denote standard errors. In all panels, the atoms are equally initialized at two leftmost sites with phase $\phi = \pi$, and the intercell hopping rate is $K/h = 0.4\,\mathrm{kHz}$.

controlled phase, and we hope this initialization is closely approximate to the theoretical distribution from the nonlinear edge state under the strong interactions. As illustrated in Fig. S5, the population dynamics reaches a stationary state for the initialized in-phase population over a certain interaction range, in compared to the unstable oscillation for the initialized out-of-phase population. This indicates that the initial in-phase equal occupation at two leftmost sites is more approaching to the theoretical population distribution determined by the nonlinear edge state under the strong interactions rather than the initialized out-of-phase population.

On the other hand, although the stationary states are not reached in many cases, the enhanced localization of atoms at two leftmost sites observed in the population dynamics illustrates that the initial population engineered in the experiment is approaching to the distribution determined by the eigenmodes of nonlinear edge states under the strong interactions rather than the

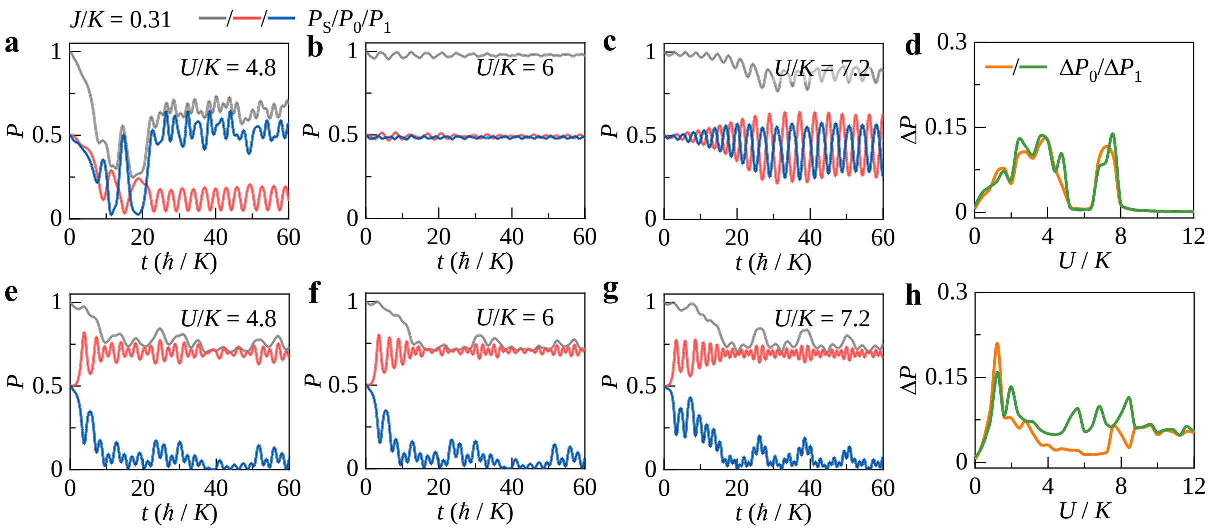

FIG. S5. Numerical calculation of population dynamics of atoms in the topological trimer array with $J/K = 0.31$ under different interactions U/K at the long time for the initial phases $\phi = 0$ in **a**-**c** and $\phi = \pi$ in **e**-**g**, where the atoms are initialized at two leftmost sites with the equal weight. P_S is the normalized population of atoms at two leftmost sites, and P_0 and P_1 are the population at the sites $n = 0$ and 1, respectively. The averaged deviation from the mean population in each of two leftmost sites ΔP_0 and ΔP_1 as a function of U/K for $\phi = 0$ in **d** and for $\phi = \pi$ in **h**, where the average is performed over the time range from $t_1 = 20\hbar/K$ to $t_2 = 60\hbar/K$.

cases for noninteraction or weak interactions.

Nonlinear edge states that bifurcate from their linear counterparts

Following Refs. [1–4], the interactions of atoms in momentum lattices can be captured by the nonlinear Schrödinger equation

$$i\frac{\partial \phi_n}{\partial t} = \sum_m H_{mn}\phi_m + U\left(2 - |\phi_n|^2\right)\phi_n, \tag{S4}$$

where ϕ_n is the normalized complex amplitude for the discrete momentum state, and H_{mn} is the matrix element of the Hamiltonian in Eq. (1) of the main text associated with momentum states p_m and p_n. We assume the solution of Eq. (S4) has the ansatz $\phi_n = u_n \exp(-iEt)$, and Eq. (S4) can be rewritten as

$$Eu_n = \sum_m H_{mn}u_m + U\left(2 - |u_n|^2\right)u_n. \tag{S5}$$

Clearly, if the mean-field interaction energy U is negligible, Eq. (S4) reduces into its linear counterpart, and the Hamiltonian is exactly same as Eq. (1) in the main text.

Generally, Eq. (S5) can be numerically solved by using the Newton iteration method [5]. The theoretical results are shown in Fig. S6a, in which the density $\sum_n |u_n|^2$ means the number of atoms at the nonlinear edge states. As expected, we have two families of nonlinear edge states, one bifurcates from the in-phase linear edge state (red curve), and the other bifurcates from the out-of-phase linear edge state (blue curve). Here, the bifurcation means that the nonlinear edge state reduces into its linear counterpart with the decrease of its density, which reflects that the nonlinear edge state originates from its linear counterpart. The propagation constant (i.e., energy E) of the nonlinear edge states deceases, across the linear band gap, and even enters into the bulk band to interact with the bulk states. The theoretical result in Fig. S6a shows that the excitation of the nonlinear edge states is thresholdless, however, the formation of nonlinear edge states has a threshold in the real experiment [5–7].

It is significant to test the stability of the nonlinear edge states shown in Fig. S6a. To this regard, we perturb the solution u_n in the form

$$\phi_n = [u_n + v_n \exp(\beta t) + w_n^* \exp(\beta^* t)] \exp(-iEt), \tag{S6}$$

where $v_n, w_n \ll 1$ are small perturbations, and β is the perturbation growth rate that can be complex. Inserting Eq. (S6) into Eq. (S4) and linearizing it around stationary solution u_n, we arrive at the linear eigenvalue problem,

$$i\beta v_n = +\sum_m H_{mn}v_m + [2U(1 - |u_n|^2) - E]v_n - U|u_n|^2 w_n, \tag{S7}$$

$$i\beta w_n = -\sum_m H_{mn}w_m - [2U(1 - |u_n|^2) - E]w_n + U|u_n|^2 v_n. \tag{S8}$$

Solving Eqs. (S7) and (S8) using a standard eigenvalue solver, we obtain the dependence of the perturbation growth rate β on the energy E for a given family of the

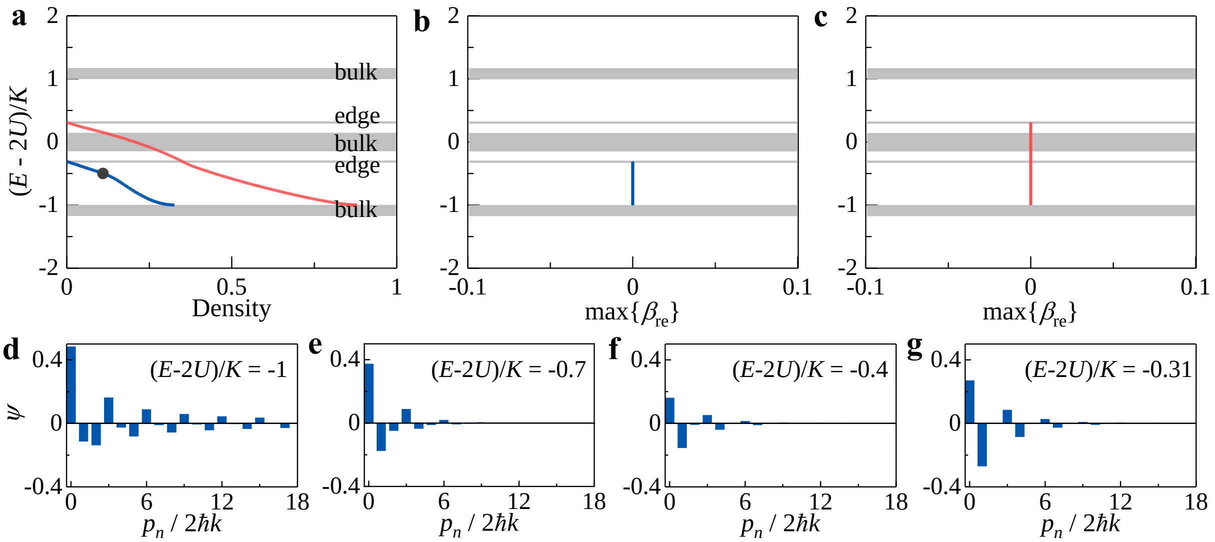

FIG. S6. **a**, Nonlinear state families bifurcating from the linear edge states of topological trimer array with $J/K = 0.31$ for $U/K = 4$. Density represents the number of atoms in the nonlinear edge state $\sum_n |u_n|^2$. The energy E/K in the vertical axial is shifted with $-2U/K$, which is an energy shift in Eq. (S4) in compared to the optical nonlinearity, and then the nonlinear edge states return back their linear counterparts when the density of the state decreases to 0. The red and blue curves bifurcate from the in-phase and out-of-phase linear edge states, respectively. **b** and **c**, Maximum growth rates of the perturbation $\beta_{\rm re}$ in **b** and **c** for the nonlinear edge states indicated with the blue and red curves in **a**. Because the maximum real part of $\beta_{\rm re}$ is always zero, two families are stable. **d-f**, The distribution of eigen wavefunction of the nonlinear edge state ψ from the blue curve in **a** with the energy E/K given in each panel. The y axis tick values are fixed and authentic, and cannot be normalized. **g**, Eigenmodes of the linear edge state bifurcating from which the nonlinear edge state (blue curve) establishes. The energy is $(E - 2U)/K = -0.31$. The y axis tick values are arbitrary, and can be scaled and normalized.

nonlinear states. If $\beta_{\rm re} \leq 0$ for all possible perturbations, the corresponding nonlinear state u_n is linearly stable. In the presence of small perturbations, it will exhibit only small amplitude oscillations upon evolution. Otherwise, if at least one perturbation mode has $\beta_{\rm re} > 0$, the nonlinear state is unstable and will decay in the course of propagation.

In Fig. S6b and c, we show the the linear stability analysis, which corresponds to the nonlinear edge states shown in Fig. S6a. Clearly, all the nonlinear edge states are stable, since the maximum real part of the growth rate for both kinds of nonlinear edge states is 0, i.e. $\max\{\beta_{\rm re}\} = 0$.

To illustrate the bifurcation of the nonlinear edge states from the linear edge states, we show the distributions of eigen wavefunctions of the nonlinear edge states in Figs. S6d-f, and these states are picked from the blue curve in Fig. S6a with varying energy E/K. Since the nonlinear edge states are eigen-solutions of Eq. (S4), the y axis tick values are authentic and fixed in Figs. S6d-f. The eigen wavefunctions of the nonlinear edge states cannot be normalized. While the eigen wavefunction of the linear edge state in Fig. S6g can be scaled arbitrarily, since it is an eigen-solution of the linear version of Eq. (S4). Therefore, the y axis tick values in Fig. S6g is arbitrary and not fixed, but the population proportion is consistent with the out-of-phase population distribution in the left side of the linear topological trimer array in Fig. 1c of the main text. When the value of $(E - 2U)/K$ changes from -1 to -0.4, the profile of the nonlinear edge state in Fig. S6f is approaching to that of the linear edge state with $(E - 2U)/K = -0.31$ in Fig. S6g. The nonlinear edge state ultimately reduces into the linear edge state completely, if the value of $(E - 2U)/K$ coincides with that of the linear edge state. This demonstrates that the nonlinear edge state originates from its linear counterpart.

Robustness of the nonlinear edge state

To check the robustness of the nonlinear edge state, we have to set a defect or make a disorder to the outermost sites, at which the nonlinear edge state locates. However, the strength of the defect or disorder should not be too big, or it will be beyond the protection of the band gap. Since the defect or disorder will affect the intracell hopping rate J, we manually increase and decrease the coupling strength between the two leftmost sites by 10%, and track the evolution of one nonlinear edge state in this perturbed trimer array. We find two kinds of nonlinear edge states are robust to the perturbation applied in the coupling strength. For example, for one nonlinear edge state from the blue curve in Fig. S6a, we demonstrate its robustness by comparing the evolution of the nonlinear edge state in the unperturbed and perturbed trimer arrays in Fig. S7. We show the evolution of the nonlinear edge state for $E/K = 7.5$ and $U/K = 4$ in the

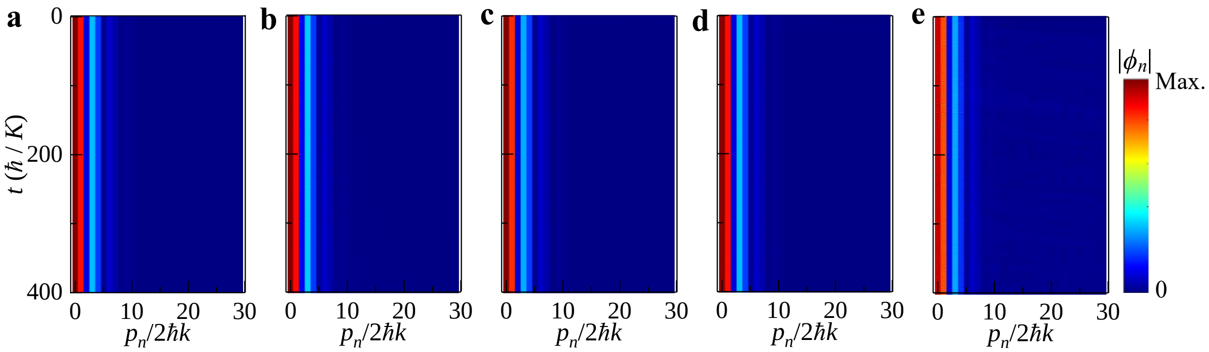

FIG. S7. Robustness of the nonlinear edge state for $E/K = 7.5$ and $U/K = 4$, i.e. $(E-2U)/K = -0.5$ in the trimer array with $J/K = 0.31$, which corresponds to the black dot on the blue curve (out-of-phase) in Fig. S6a. **a**, Evolution of the nonlinear edge state in the unperturbed trimer array. **b** and **c**, Evolution of the nonlinear edge state in the perturbed trimer arrays for different hopping rates $1.1J$ in **b** and $0.9J$ in **c** between the two leftmost sites. **d** and **e**, Evolution of the nonlinear edge state with a small perturbation that is imposed on the input nonlinear edge state in **d** and the non-diagonal elements of the Hamiltonian in **e**. In all panels, we show the absolute value of the field of the state.

unperturbed trimer array with $J/K = 0.31$ in Fig. S7a, and demonstrates that the stability of the nonlinear edge state during the evolution. When the coupling strength between the first and second sites on the left side of trimer array is increased by 10% and decreased by 10%, respectively, and we show that the nonlinear edge state can maintain its profile during the evolution in Fig. S7b and c.

In addition to the above method, we provide another two methods to check the robustness of the nonlinear edge state. First, we add a perturbation to the input nonlinear edge state via $\psi \to (1 + 0.2\delta_p)\psi$, where δ_p is a vector with random numbers in the range $[-0.5, 0.5]$, and the strength of the perturbation is 10% of the nonlinear edge state in amplitude. The evolution of the nonlinear edge state with the perturbation imposed on the ψ is shown in Fig. S7d. Second, we add a perturbation to the non-diagonal elements of the Hamiltonian, which is a matrix and its diagonal elements are controlled by the nonlinearity. The perturbation is imposed on the non-diagonal elements by $H_{\text{lin}} \to (1 + 0.2\delta_H)H_{\text{lin}}$, where δ_H is a matrix with random numbers in the range $[-0.5, 0.5]$, and H_{lin} is the Hamiltonian by removing the diagonal elements. The evolution of the nonlinear edge state with the perturbation imposed on the H_{lin} is shown in Fig. S7e, and the density fluctuates a bit during the evolution due to the perturbation to the Hamiltonian (i.e. the system). The results in Fig. S7d and e clearly illustrate that the nonlinear edge state is robust since it survives from the perturbation.

[1] F. A. An, E. J. Meier, J. Ang'ong'a, and B. Gadway, Correlated Dynamics in a Synthetic Lattice of Momentum States, Phys. Rev. Lett. **120**, 040407 (2018).

[2] F. A. An, E. J. Meier, and B. Gadway, Engineering a Flux-Dependent Mobility Edge in Disordered Zigzag Chains, Phys. Rev. X. **8**, 031045 (2018).

[3] F. A. An, B. Sundar, J. P. Hou, X.-W. Luo, E. J. Meier, C. W. Zhang, K. R. A. Hazzard, and B. Gadway, Nonlinear Dynamics in a Synthetic Momentum-State Lattice, Phys. Rev. Lett. **127**, 130401 (2021).

[4] T. Chen, D. Z. Xie, B. Gadway, and B. Yan, A Gross-Pitaevskii-equation description of the momentum-state lattice: roles of the trap and many-body interactions, Preprint at https://doi.org/10.48550/arXiv.2103.14205 (2021).

[5] B. Ren, A. A. Arkhipova, Y. Zhang, Y. V. Kartashov, H. Wang, S. A. Zhuravitskii, N. N. Skryabin, I. V. Dyakonov, A. A. Kalinkin, S. P. Kulik, V. O. Kompanets, S. V. Chekalin, and V. N. Zadkov, Observation of nonlinear disclination states, Light Sci. Appl. **12**, 194 (2023).

[6] Y. V. Kartashov, A. A. Arkhipova, S. A. Zhuravitskii, N. N. Skryabin, I. V. Dyakonov, A. A. Kalinkin, S. P. Kulik, V. O. Kompanets, S. V. Chekalin, L. Torner, and V. N. Zadkov, Observation of Edge Solitons in Topological Trimer Arrays, Phys. Rev. Lett. **128**, 093901 (2022).

[7] H. Zhong, V. O. Kompanets, Y. Zhang, Y. V. Kartashov, M. Cao, Y. Li, S. A. Zhuravitskii, N. N. Skryabin, I. V. Dyakonov, A. A. Kalinkin, S. P. Kulik, S. V. Chekalin, and V. N. Zadkov, Observation of nonlinear fractal higher order topological insulator, Light Sci. Appl. **13**, 264 (2024).
